# cAMP/PKA Signaling Modulates Mitochondrial Supercomplex Organization

**DOI:** 10.3390/ijms23179655

**Published:** 2022-08-25

**Authors:** Anna Signorile, Consiglia Pacelli, Luigi Leonardo Palese, Arcangela Santeramo, Emilio Roca, Tiziana Cocco, Domenico De Rasmo

**Affiliations:** 1 Department of Basic Medical Sciences, Neurosciences and Sense Organs, University of Bari “Aldo Moro”, 70124 Bari, Italy; 2 Department of Clinical and Experimental Medicine, University of Foggia, 71122 Foggia, Italy; 3 Institute of Biomembranes, Bioenergetics and Molecular Biotechnology, National Research Council, 70124 Bari, Italy

**Keywords:** mitochondria, cAMP/PKA, complex I, mitochondrial supercomplexes, NDUFS4

## Abstract

The oxidative phosphorylation (OXPHOS) system couples the transfer of electrons to oxygen with pumping of protons across the inner mitochondrial membrane, ensuring the ATP production. Evidence suggests that respiratory chain complexes may also assemble into supramolecular structures, called supercomplexes (SCs). The SCs appear to increase the efficiency/capacity of OXPHOS and reduce the reactive oxygen species (ROS) production, especially that which is produced by complex I. Studies suggest a mutual regulation between complex I and SCs, while SCs organization is important for complex I assembly/stability, complex I is involved in the supercomplex formation. Complex I is a pacemaker of the OXPHOS system, and it has been shown that the PKA-dependent phosphorylation of some of its subunits increases the activity of the complex, reducing the ROS production. In this work, using in ex vivo and in vitro models, we show that the activation of cAMP/PKA cascade resulted in an increase in SCs formation associated with an enhanced capacity of electron flux and ATP production rate. This is also associated with the phosphorylation of the NDUFS4 subunit of complex I. This aspect highlights the key role of complex I in cellular energy production.

## 1. Introduction

In human cells, the ATP produced by the mitochondrial oxidative phosphorylation (OXPHOS) system covers the major part of cellular requirement; the remainder is provided by glycolysis. The oxidation of NADH or FADH_2_ by mitochondrial respiratory chain complexes couples the transfer of electrons to oxygen with pumping of protons across the inner mitochondrial membrane (IMM), thus generating a transmembrane proton gradient which drives ATP formation by complex V [1,2]. Evidence, mainly based on structural analysis, suggests that respiratory complex I (CxI), III (CxIII) and IV (CxIV), can also assemble into supramolecular structures called supercomplexes (SCs). The SCs contain respiratory complexes in various stoichiometry, such as CxI + CxIII_2_, CxIII_2_ + CxIV, and CxI + CxIII_2_ + CxIV [3,4]. The assembly of these complexes is also called “respirasome” because, together with the mobile transporters coenzyme Q and cytochrome c, it works as a single functional unit that transfers electrons and simultaneously forms the electrochemical gradient through the IMM [3,5]. Evidence suggests that SCs play a role in the electron flux of the respiratory chain [6,7] and also reduce the production of reactive oxygen species (ROS) [8,9,10]. A mutual regulation between complex I and SCs has been observed, since SCs organization is important for complex I assembly/stability [11,12,13,14,15] and complex I has an important role in the formation of the supercomplexes [13].

Complex I is the entry point of the reducing equivalents. It catalyzes the electron transfer from NADH to ubiquinone [1,2] and plays a central role in OXPHOS efficiency and capacity [16]. In mammals, in addition to 14 conserved subunits of the catalytic core, complex I has 31 supernumerary subunits. Seven subunits are encoded by the mitochondrial DNA and 38 by nuclear genes [17,18]. The function of the supernumerary subunits is largely unknown. Some are involved in the assembly of the complex, its regulation and ancillary functions [18,19]. Complex I, together with complex III, is the major site of oxygen superoxide formation [20], and its functional capacity is regulated by a variety of regulatory factors/events acting at the expression and post-translational levels [2,17,21,22]. Indeed, several subunits of complex I can be acetylated, glutathionylated [23], and phosphorylated [22].

The cAMP/PKA pathway has an important role in the regulation of complex I [24] by activating complex I biogenesis [25,26] and by increasing its Vmax and decreasing complex I-dependent ROS production [27,28,29]. In cell cultures, the activation of cAMP/PKA pathway, by permeant derivative of cAMP or by the β-agonist isoproterenol, promotes the phosphorylation of NDUFS4 protein, under the conditions in which it activates complex I and reverses ROS accumulation [27,28]. In addition, the mitochondrial import of the NDUFS4 protein and its assembly in complex I are stimulated by PKA-mediated phosphorylation [30]. It has been proposed that the activity of complex I can be promoted by the phosphorylation-dependent import of newly synthesized NDUFS4 protein that, once imported into mitochondria, can be assembled in complex I [31,32] via exchange with the “aged” subunit in the complex [29]. Interestingly, it is reported that mutations in the NDUFS4 gene, resulting in the disappearance of the protein, were associated with combined enzymatic complex I and complex III deficiency, suggesting an involvement in SCs formation [33]. On the other hand, studies showed that the SCs formation reduced the ROS production [34] and, in particular, the complex I-dependent ROS production [8,9]. Despite the well-studied role of cAMP/PKA pathway on complex I activity and its dependent ROS production, no information is present on the role of this signaling in the regulation of SCs formation. Herein, we show that, in H9c2 cell cultures and in isolated rat liver mitochondria (RLM), the activation of cAMP/PKA cascade resulted in an increase in SCs formation associated with an increased capacity of the electron flux and ATP production rate, probably promoted by phosphorylation of NDUFS4 subunit.

## 2. Results

### 2.1. Effect of Isoproterenol Treatment of H9c2 Cells on Mitochondrial Respiratory Complex Activities and SCs Organization

We reported that β-agonist isoproterenol, which induces an increase in the cAMP level, is associated with the PKA-dependent phosphorylation of the NDUFS4 subunit of complex I [28].

**Figure 1:**
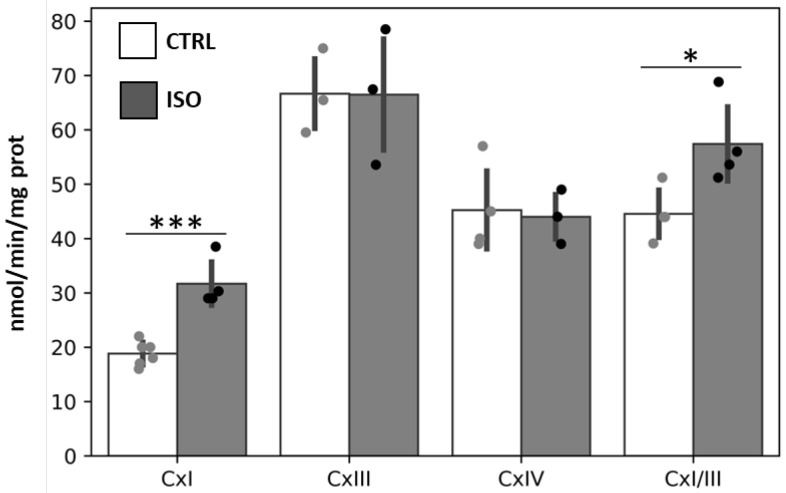
Effect of isoproterenol treatment on activity of mitochondrial respiratory chain complexes. The H9c2 cell cultures were incubated for 30 min in absence (CTRL) or in the presence of 1 µM isoproterenol (ISO). The activities of complexes I (CxI), III (CxIII), IV (CxIV) and I + III (CxI/III) were measured spectrophotometrically. The scatter bar graphs represent the means ± standard deviation (SD). CTRL vs. ISO, * *p* < 0.05, *** *p* < 0.001, Student’s *t*-test.

Isoproterenol (ISO) treatment of H9c2 cell cultures resulted in a significant stimulation of the complex I (NADH-UQ oxidoreductase) and complex I/III activities (NADH-cytochrome c oxidoreductase), without any effect on the activities of complex III (ubiquinol–cytochrome c oxidoreductase) and complex IV (cytochrome c oxidase) with respect to control cells (CTRL) (Figure 1).

**Figure 2:**
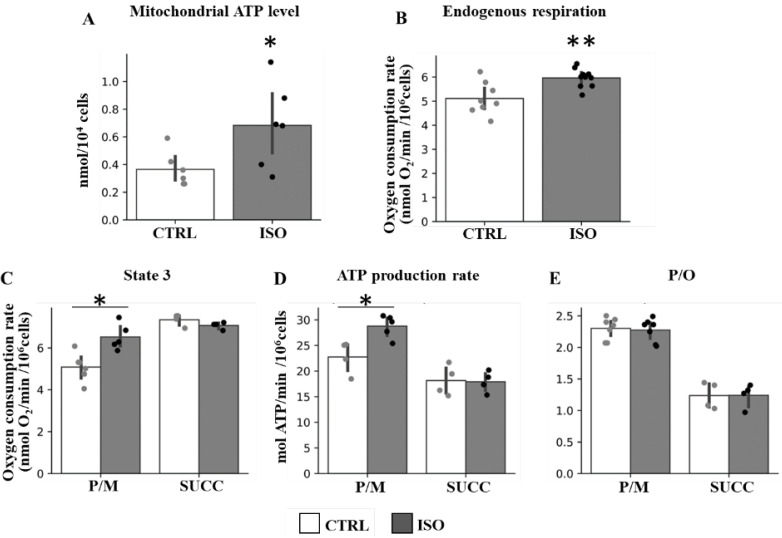
Effect of isoproterenol treatment on oxygen consumption rates, ATP level, ATP production rate and efficiency. The H9c2 cell cultures were incubated in absence (CTRL) or in the presence of isoproterenol (ISO). (**A**) The scatter bar graphs represent the mitochondrial ATP level determined as result of total ATP level minus ATP level in the presence of oligomycin. (**B**) The scatter bar graphs represent the oxygen consumption rates measured in intact cells (endogenous). (**C**) The scatter bar graphs represent the oxygen consumption rates in digitonin-permeabilized cells supplemented with ADP and pyruvate plus malate (P/M) or succinate (SUCC) as respiratory substrates (State 3). (**D**) The scatter bar graphs represent the mitochondrial ATP production rates measured in the presence of P/M or SUCC. (**E**) The scatter bar graphs represent the P/O ratio measured in the presence of P/M or SUCC. The data represent the means ± SD of at least five different experiments except for succinate (four determinations). ISO vs. CTRL, * *p* < 0.05, ** *p* < 0.01, Student’s *t*-test.

Accordingly, stimulation of the endogenous respiration and mitochondrial ATP level, as well as of state 3 respiration and ATP production rates, supported only by NAD-linked substrates (pyruvate plus malate, P/M) was observed after isoproterenol treatment of H9c2 cells (Figure 2). No difference in state 3 respiration and ATP production rates was observed in the case of succinate-supporting respiration (Figure 2). Instead, the P/O ratio remained unchanged in both NAD-linked and succinate supporting respiration (Figure 2).

**Figure 3:**
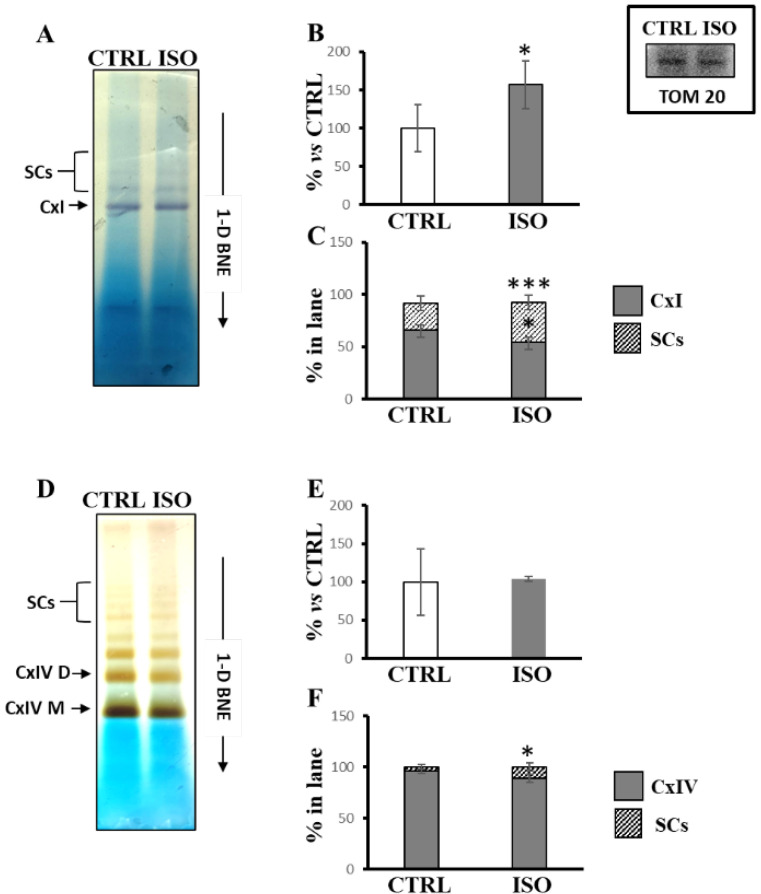
Isoproterenol increased the activities of complex I and complex IV in supercomplexes. The H9c2 cell cultures were incubated for 30 min in absence (CTRL) or in the presence of 1 µM isoproterenol (ISO). Mitochondrial solubilized proteins were prepared as described in Materials and Methods and separated by 1D-BNE PAGE followed by in-gel activity assay. (**A**) Representative image of in gel-activity assay for complex I. (**B**) The histograms represent the percentage value of arbitrary densitometric units (ADU) of complex I activity-stained bands of ISO treated cells with respect to CTRL cells. (**C**) The histograms represent the percentage of ADU of complex I activity-stained bands referring to free complex I (CxI) and supercomplexes (SCs) in each lane. (**D**) Representative image of in-gel activity assay for complex IV. (**E**) The histograms represent the percentage value of ADU of complex IV activity-stained bands of ISO treated cells with respect to CTRL cells. (**F**) The histograms represent the percentage of ADU of complex IV activity-stained bands referring to free complex IV (CxIV) and SCs in each lane. The values are the means ± SD of three different experiments. (ISO vs. CTRL, * *p* < 0.05, *** *p* < 0.001, Student’s *t*-test). In the inset, a representative image of immunoblotting with antibody against TOM20 performed for the protein loading control of CTRL and ISO samples. For other details, see under Material and Methods.

Notably, after blue native electrophoresis (1D-BNE) of H9c2 cell lysate, the in-gel colorimetric assay for analysis of complex I activity revealed three colored bands, of which one was ascribable to free complex I (CxI) and two to SCs containing complex I in both untreated and ISO-treated cells (Figure 3A). According to spectrophotometric measurements, the densitometric analysis showed an increase in total in-gel activity (sum of the three bands) in ISO-treated cells with respect to control cells (Figure 3B). In particular, the specific distribution of in-gel activity changed in ISO-treated cells with respect to untreated cells, showing that the percentage of in-gel activity augmented for supercomplexes containing complex I and decreased for the free complex I (Figure 3C). In addition, in-gel colorimetric assay for analysis of complex IV activity revealed, as also reported by other authors [35], several bands ascribable to monomeric complex IV (CxIV M), dimeric complex IV (CxIV D), and several SCs containing complex IV (Figure 3D). While no change was observed for the total in-gel activity assay (Figure 3E), ISO treatment resulted in increased color intensity in supercomplexes containing complex IV (Figure 3F).

**Figure 4:**
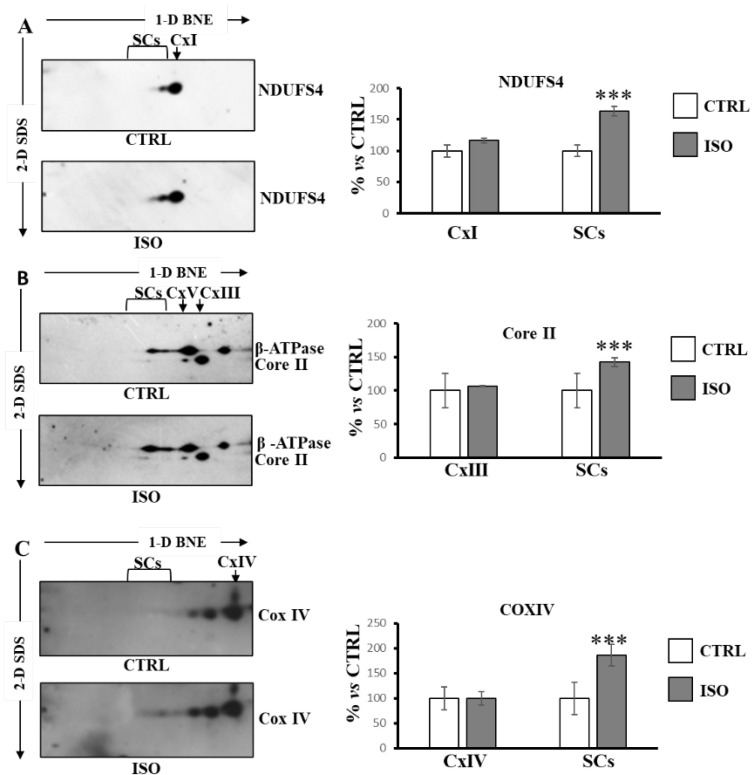
Isoproterenol increased supercomplexes containing complex I, complex III and complex IV. The H9c2 cell cultures were incubated for 30 min in absence (CTRL) or in the presence of isoproterenol (ISO). Mitochondrial solubilized proteins were separated by 1D-BNE/2D-SDS-PAGE and transferred into nitrocellulose membrane, followed by immunoblotting analysis with specific antibodies. (**A**) Representative images of immunoblotting with antibody against NDUFS4. (**B**) Representative images of immunoblotting with antibodies against Core II and β-ATP synthase. (**C**) Representative images of immunoblotting with antibody against Cox IV. (**A**–**C**) the histograms represent the percentage of ADU of isoproterenol-treated cells (ISO) with respect to untreated cells (CTRL) of immuno-revealed spots in free complexes (CxI, CxIII, CxIV) and in supercomplexes (SCs). The values are the means ± SD of three different experiments. (ISO vs. CTRL, *** *p* < 0.001, Student’s *t*-test). For more details, see under Materials and Methods.

Next, we performed BNE/SDS PAGE of H9c2 cell lysate followed by Western blotting for structural analysis of SCs organization. The antibodies against NDUFS4 for complex I, Core II for complex III, beta for ATP synthase and CoxIV for complex IV, were used sequentially. The beta subunit antibody for ATP synthase was used for the alignment of complexes. As shown in Figure 4, according to the functional data, ISO treatment promoted an increase in SCs containing complex I (NDUFS4 subunit) (Figure 4A), as well as an increase in SCs containing complex III (Core II subunit) (Figure 4B) and complex IV (Cox IV subunit) (Figure 4C).

### 2.2. Effect of PKA Signaling on the Phosphorylation-Dependent Mitochondrial Import and Assembly of the NDUFS4 Subunit

NDUFS4 is reported to be a target for PKA and, in particular, the protein has been found to be phosphorylated in mammalian cells [36] and in rabbit reticulocyte lysate (RRL) [30] after incubation with PKA. Thus, we analyzed the effect of PKA-dependent phosphorylation on the import and incorporation of NDUFS4 subunit in free complex I and SCs.

**Figure 5:**
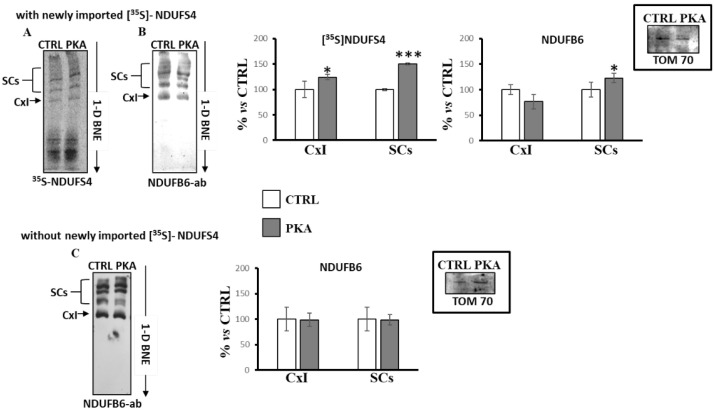
PKA addition to the mitochondrial import experiments increased the incorporation of [^35^S]-Met labeled NDUFS4 in supercomplexes. Mitochondrial import experiments were performed in absence (CTRL) and in the presence of PKA (1 unit per 10 μg mitochondrial proteins) plus 7.5 mM sodium fluoride. After 60 min incubation at 30 °C, mitochondria were spun down from the import mixture after trypsin treatment (1 μg/50 μg mitochondrial proteins, 35 min on ice). The solubilized pellets were analyzed by 1D-BNE and transferred into nitrocellulose. (**A**) Representative image of autoradiography of import experiment with RRL translation mixtures containing the newly synthesized [^35^S]-Met labeled NDUFS4 protein. (**B**) Representative image of immunoblotting analysis with a specific antibody against the NDUFB6 subunit of complex I of import experiment with RRL translation mixtures containing the newly synthesized [^35^S]-Met labeled NDUFS4 protein. (**C**) Representative image of immunoblotting analysis with a specific antibody against the NDUFB6 subunit of complex I of import experiment with RRL translation mixtures without the newly synthesized [^35^S]-Met labeled NDUFS4 protein. The histograms represent the percentage of ADU of PKA-treated samples with respect to untreated samples (CTRL) of radioactive bands ([^35^S]-Met NDUFS4) and immuno-revealed bands (NDUFB6) in free complex I (CxI) and in SCs. The values are the means ± SD of three different experiments. (PKA vs. CTRL, * *p* < 0.05, *** *p* < 0.001, Student’s *t*-test). The insets represent the loading controls performed using TOM 70 antibody after the SDS-PAGE. For more details, see under Materials and Methods.

RLM were incubated with [^35^S]-Met labeled NDUFS4 in the absence (CTRL) or in the presence of catalytic subunit of PKA (PKA). After incubation, the mitochondria were pelleted and processed by 1D-BNE, followed by autoradiography (Figure 5A) or immunoblotting analysis (Figure 5B). Figure 5A shows that the new imported [^35^S]-Met labeled NDUFS4, in absence of PKA, was incorporated in different bands ascribable to free complex I and some higher molecular weight bands corresponding to SCs containing complex I. In the presence of PKA, the incorporation of new imported [^35^S]-Met labeled NDUFS4 increased in both free complex I and SCs containing complex I (Figure 5A). Western blotting analysis of the same samples using an antibody against NDUFB6 subunit of complex I (Figure 5B) revealed that, in the presence of PKA, the amount of complex I increased in SCs, but tendentially decreased in free complex I, even if the data were not statistically significant (Figure 5B). The same experiments (Figure 5C), performed in RLM without the presence of the new imported [^35^S]-Met labeled NDUFS4, showed that the addition of PKA did not alter the distribution of the free complex I or complex I in SCs evaluated with the antibody against NDUFB6 subunit (Figure 5C), thus suggesting a key role of PKA-dependent phosphorylation of NDUFS4 in SCs formation.

**Figure 6:**
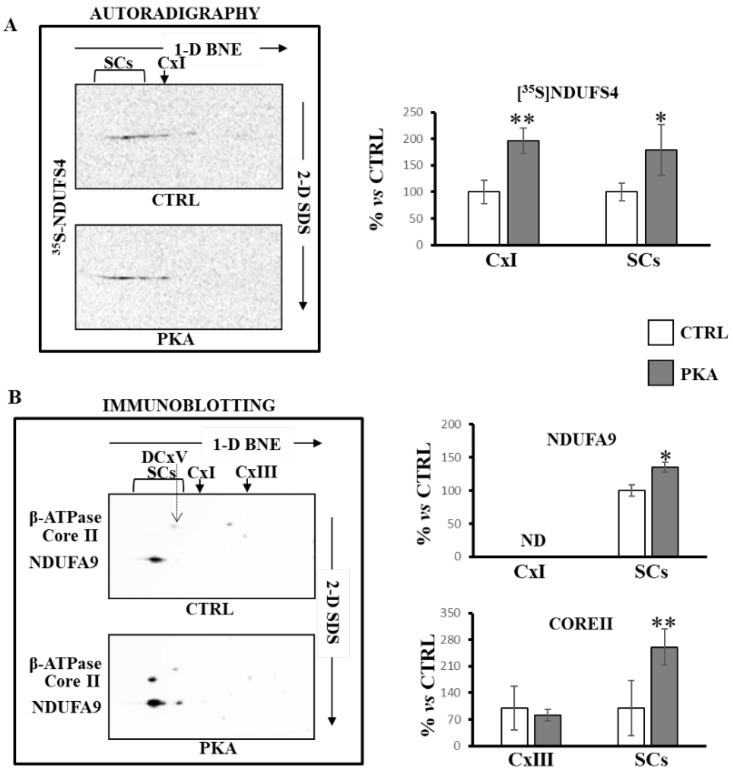
PKA addition to mitochondrial import experiments increased supercomplexes containing complex I and complex III. RRL translation mixture containing the newly synthesized [^35^S]-Met labeled NDUFS4 protein was added to the import mixture containing RLM in absence (CTRL) and in the presence of PKA. After 60 min incubation at 30 °C, mitochondria were spun down. The solubilized pellets were analyzed by 1D-BNE/2D-SDS PAGE. (**A**) Representative images of autoradiography. The histograms represent the percentage of ADU of autoradiography revealed spots ([^35^S]-Met NDUFS4) in free complex I (CxI) and in supercomplexes (SCs) of PKA-treated samples with respect to untreated cells (CTRL). The complex alignment of radioactive spots was performed by using first dimension radioactive bands of the same lanes. (**B**) Representative images of immunoblotting analysis with antibodies specified in the figure. The histograms represent the percentage of ADU of PKA-treated samples with respect to untreated cells (CTRL) of immuno-revealed spots by antibodies against NDUFA9 and CORE II in free complex I (CxI) and complex III (CxIII) and in supercomplexes (SCs). The complex alignment of immuno-revealed spots was performed by using the β-subunit antibody revealing the dimeric form of ATP synthase (DCxV). The values are the means ± SD of three different experiments. (PKA vs. CTRL, * *p* < 0.05, ** *p* < 0.01, Student’s *t*-test). For more details, see under Materials and Methods.

These results were confirmed when mitochondria, incubated with [^35^S]-Met labeled NDUFS4 in the absence or in the presence of PKA, were subjected to BNE/SDS PAGE and then transferred on nitrocellulose (Figure 6). The autoradiography of the second dimension (2D-SDS) showed an enrichment of [^35^S]-Met labeled NDUFS4 in PKA-treated mitochondria both in free complex I and in supercomplexes containing complex I (Figure 6A). The Western blotting analysis with antibody against complex I (NDUFA9) and complex III (Core II) subunits again revealed that NDUFS4 and PKA promoted the SCs formation (Figure 6B).

### 2.3. Effect of PKA Signaling and Import of NDUFS4 Subunit on the Activity of Complex I, Complex III, Complex I/III and Mitochondrial Respiration Rates

**Figure 7:**
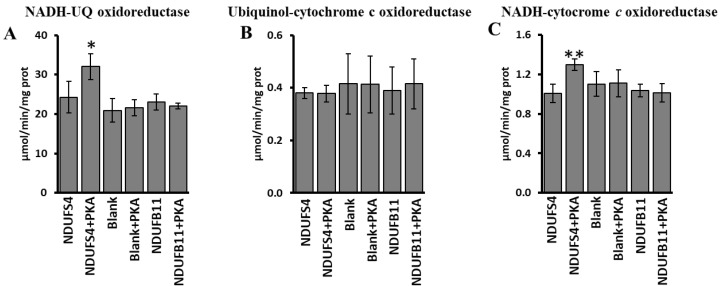
PKA addition to mitochondrial import experiments increased the activity of complex I and complex I/III. RRL translation mixtures containing newly synthesized [^35^S]-Met labeled NDUFS4 protein or [^35^S]-Met-labeled NDUB11 protein or no protein present (Blank) were added to the import mixture containing RLM in absence and in the presence of PKA. After 60 min incubation at 30 °C, mitochondria were spun down from the import mixture and used for spectrophotometric analysis. (**A**) Activity of complex I (NADH-UQ oxidoreductase). (**B**) Activity of complex III (ubiquinol-cytochrome *c* oxidoreductase). (**C**) Activity of complex I/III (NADH-cytochrome *c* oxidoreductase). The values are the means ± SD of three different experiments. (NDUFS4 + PKA vs. NDUFS4, * *p* < 0.05, ** *p* < 0.01, Student’s *t*-test). For more details, see under Materials and Methods.

To determine the specific functional effect of PKA-phosphorylated NDUFS4 in promotion of SCs organization, the activity of complex I, complex III, complex I/III was evaluated in RLM after import of the [^35^S]-Met labeled NDUFS4 or [^35^S]-Met labeled NDUFB11, another subunit of complex I, or in the absence of newly synthesized imported proteins (Figure 7). All conditions were carried out in the absence or in the presence of PKA (Figure 7). As shown in Figure 7, an increase in the activities of complex I (NADH-UQ oxidoreductase) (panel A) and complex I/III (NADH-cytochrome c oxidoreductase) (panel C) was observed when [^35^S]-Met labeled NDUFS4 was imported in the presence of PKA. No effect by [^35^S]-Met NDUFS4 import and PKA treatment was observed on complex III activity (Ubiquinol-cytochrome c oxidoreductase) (Figure 7B). No changes were observed in the case of control (Blank) experiments, in which no subunits were imported, or in the case in which the [^35^S]-Met labeled NDUFB11 was imported (Figure 7A–C), thus confirming the main role of the NDUFS4 phosphorylation.

**Figure 8:**
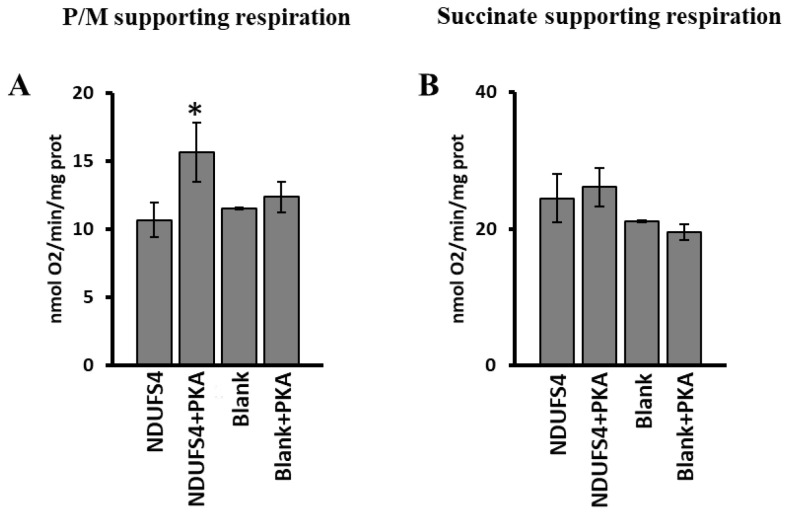
PKA addition to mitochondrial import experiments increased the complex I-supporting respiration. RRL translation mixtures containing newly synthesized [^35^S]-Met-labeled NDUFS4 protein or no protein present (Blank) were added to the import mixture containing RLM in absence and in the presence of PKA. After 60 min incubation at 30 °C, mitochondria were spun down from the import mixture and used to measure the oxygen consumption rates. (**A**) Oxygen consumption rates using pyruvate/malate (P/M) as substrate. (**B**) Oxygen consumption rates using succinate as substrate. The values are the means ± SD of three different experiments. (NDUFS4 + PKA vs. NDUFS4, * *p* < 0.05, Student’s *t*-test). For more details, see under Materials and Methods.

The RLM incubated with [^35^S]-Met labeled NDUFS4 or without it (Blank), in the presence and in the absence of PKA, were also used to evaluate the respiration rates. A significant increase in pyruvate/malate-dependent oxygen consumption was observed in the case of [^35^S]-Met NDUFS4 import in the presence of PKA (Figure 8A) compared to the import of NDUFS4 import without PKA addition. No effect was observed in RLM incubated without the [^35^S]-Met labeled NDUFS4 and PKA (Blank) (Figure 8A). The use of succinate as substrate did not show any change in the rate of oxygen consumption in all the experimental conditions used (Figure 8B).

## 3. Discussion

The supernumerary subunits present in the mammalian complex I have a critical role in the complex’s function, regulation, stability, assembly, and protection against oxidative stress [22]. Some of these are post-translationally modified, resulting in a modulation of complex activity [22,23,28]. Among them, the cAMP/PKA-dependent phosphorylation of the NDUFS4 supernumerary subunit is one of the most studied and discussed post-translational modifications [36,37,38,39,40,41], which primes the complex I activity and reduces the ROS production [28,29].

Complex I can also form, with complex III and complex IV, supramolecular organizations called respiratory supercomplexes [3]. The proportion of the free individual complex I and the complex I in supercomplexes appears to be modulated in rodents, bovine, and zebrafish, through physiological stimuli such as the CoQH_2_/CoQ ratio [6,42]. Despite the finding on the role of cAMP in the regulation of complex I activity and biogenesis [24] and ROS formation, no information is available on the role of cAMP in the supercomplex formation and/or stabilization. In the present work, we show that cAMP signaling is able to promote the supercomplex formation likely associated with the phosphorylation of the NDUFS4 subunit of complex I.

We first showed that 30 min of isoproterenol treatment of H9c2 cell cultures induced an increase in endogenous and NADH-linked substrates respiration under phosphorylating conditions (state 3 respiration rate) associated with an increase in mitochondrial ATP level and ATP production rate. The total ATP production depends on the efficiency (P/O ratio) and the capacity of the respiratory chain under phosphorylating conditions [16]. The P/O ratio did not change in H9c2 isoproterenol-treated cells, ruling out a coupling effect, but an increase in state 3 respiration, resulting in an increase in mitochondrial ATP production rate, has been found. Moreover, an interesting finding comes from the observation of no significant difference in the P/O ratio values and in the state 3 respiration rates, between untreated and isoproterenol-treated cells, using succinate as respiratory substrate. These data, while ruling out the presence of a coupling effect and of a contribution of complex III or IV in the increased capacity of the respiratory chain, seem to indicate complex I is the main target of isoproterenol-induced cAMP/PKA signal in the regulation of the maximal capacity of the mitochondrial respiratory chain. According to this hypothesis, isoproterenol treatment resulted in an increase in complex I and complex I/III Vmax activities without any effect on complex III and IV activities. In agreement with this, analysis of the phosphoserine pattern of BNE/SDS PAGE of untreated and isoproterenol-treated rat liver tissue showed that complex I is the major site of PKA-dependent phosphorylation among the mitochondrial respiratory chain complexes (Supplemental Figure S1).

The observed increased I/III activity, probably depending on complex I as the rate-limiting step, is in agreement with a possible role proposed for SCs in the electrons flux organization that provides a kinetic advantage [43], preventing electron traffic jams [6,7] and minimizing ROS production [8,9,10]. In this way, we investigated supercomplexes’ organization after isoproterenol treatment. The structural analysis showed that isoproterenol enhanced the supercomplex composition formed by at least complex I, complex III, and complex IV. This was also associated with increased in-gel activity of complex I and complex IV in bands corresponding to supercomplexes.

Since in the previous studies, the effect of cAMP on complex I activity was associated with the PKA-dependent mitochondrial import of the NDUFS4 protein, to further support these data, we performed in vitro mitochondrial import experiments on the [^35^S]-Met labeled NDUFS4. In particular, the new experiments confirm the PKA-dependent increased assembly of the new imported [^35^S]-Met labeled NDUFS4 in the free complex I and, in addition, show increased incorporation in the SCs containing complex I. The increased SCs formation in the presence of PKA was revealed by the radioactivity of [^35^S]-Met labeled NDUFS4 and verified by Western blotting analysis using antibodies against a subunit of complex I and a subunit of complex III.

The supercomplex I/III has been largely studied and, in particular, pulse chase experiments have shown that a subcomplex of complex I can bind complex III and complete its maturation in the supercomplex [15]. In other words, complex III can act as a scaffold to promote the final step of complex I assembly in SCs. Moreover, it has been shown that in the absence of NDUFS4 subunit, the complex I assembly is partially abolished, resulting in the formation of a subcomplex of 830 kDa lacking the dehydrogenase module (N module) [32,44,45,46]. This is associated with a combined enzymatic complex I and complex III deficiency [33]**,** suggesting involvement in SCs formation [33]. In this regard, some studies report that, in the absence of NDUFS4 in pathological condition or knockout mice, the formation of a supercomplex (subcomplex I + complex III), is still allowed with residual dehydrogenase activity [31,32]. However, in other studies, no indication of an association of incomplete complex I with complex III has been provided [19]**;** rather, the data showed that the formation of SCs occurred after the complex I assembly was fully completed, suggesting a separate sequential processes with a certain temporal gap [19,47]. Furthermore, NDUFS4 has a role in the coordination of complex I assembly by modulating the association of the N-module [19,22] until the membrane arm is assembled and, at the same time, NDUFS4 is needed for the structural integrity of the peripheral arm [48]. It has also been proposed that the phosphorylation of the NDUFS4 protein at the C-terminus site should be allocated in a pocket insufficient to accommodate a phosphate group, and thus, a conformational change is mandatory [22]. In this regard, we examined the overlap of NDUFS4 structures in the structures of free complex I and in the structures of supercomplex (Supplemental Figure S2). Rigid structural superposition was performed as described in [49,50] using the NDUFS4 in the PDB entry 6ZKO as reference and visualized in VMD [51]. Secondary structure was determined as described in [52]. The overlap of the NDUFS4 (atomic structures from [53]) reported in the structures of free complex I with a resolution better than 3.40 Å (structures codes from [18,54,55,56,57,58,59,60]) showed an extreme similarity (Supplemental Figure S2A), which is even more significant if we consider that the structures considered concern different species (even if they are correlated in terms of all of them being mammals) and that they were obtained in different conditions. In fact, it ranges from open to closed conformations of the complex, from structures not in turnover to structures obtained by flash freezing the complex in turnover conditions, to structures linked to various substrates or inhibitors. Although an apparent small displacement with respect to most of the subunit structures reported in the PDB is observed at the C-terminus region containing the phosphorylatable serine in the case of some supercomplexes (structures coordinates from [61]) (Supplemental Figure S2B), the low resolution at which the latter have been refined does not allow definitive conclusions to be drawn. Since previous experimental data suggest that the protein is phosphorylated outside of mitochondria, and that the protein is able to be imported into mitochondria in the phosphorylated status [30]**,** priming complex I activity [29]**,** it could be proposed that the phosphorylation-dependent mitochondrial import of the NDUFS4 protein is the checkpoint in defining the assembly of NDUFS4 in the pre-existing complex I/complex III supercomplex, and thus contributes to the increase in SCs formation. In fact, in addition to “de novo” biogenesis of complex I, several subunits, including NDUFS4, belonging to the matrix moiety of the complex undergo a rapid turnover, exchanging pre-existing with newly synthesized copies [29,31] via a mechanism called dynamic exchange [31]. This agrees with the findings showing that the NDUSF4, once imported in mitochondria, can be assembled in supercomplex-containing complex I. The presence of PKA (cAMP-PKA pathway) can promote the assembly of complex I in SCs. Similar results were obtained with mitochondrial import experiment using fibroblast cell cultures in which the stimulation of the [^35^S]-Met labeled NDUFS4 mitochondrial import by the addition of cAMP resulted in increased in-gel activity of complex I at the supercomplex level (Supplemental Figure S3). Furthermore, the PKA-dependent enhancement of supercomplex formation is also associated with an increase in the activity of complex I, as already reported [28,29], and of complex I/III, as well as the P/M-supporting respiration rates, without any effect on free complex III activity. Moreover, in the same experimental conditions, performed in the absence of the newly synthesized NDUFS4 or in the presence of newly synthesized NDUFB11 subunit, which does not belong to N module, no difference was observed in the distribution of supercomplexes or in the activities of complex I and complex I/III, or in pyruvate/malate-supporting respiration rate, suggesting a role in the supercomplex formation for PKA-dependent NDUFS4 phosphorylation, even if a contribution of other phosphorylatable subunits or subunits that interact with the C-terminus of NDUFS4 cannot be excluded.

The proposed role of supercomplexes in the increased efficiency/capacity of the respiratory chain is also linked to the decrease in ROS production [8,9]. This is also in agreement with the positive effect of cAMP on complex I activity associated with a strong prevention of ROS production [28,29,62]. It worth mentioning that the NDUFS4 gene is a hotspot of mutations. Mutations in this gene are often associated with Leight syndrome and, at molecular level, result in the disappearance of the protein, decreased complex I activity, formation of a subcomplex of around 830 kDa, probably lacking the N module, and, overall, the disappearance of the positive effect of cAMP on complex I activity and ROS production [24]. Therefore, our data suggest that by targeting complex I and likely the NDUFS4 subunit, the cAMP/PKA pathway promotes supercomplex formation in order to optimize the oxidation of substrates for ATP synthesis and to reduce ROS production. Thus, the cAMP signal primes an adaptive change in mitochondrial OXPHOS capacity that could be regulated by complex I through supercomplex formation, thus highlighting the key role of this complex in cellular energy production.

## 4. Materials and Methods

### 4.1. Cell Cultures

H9c2 heart myoblast cell line, derived from embryonic rat heart, was purchased from American Type Culture Collection (Manassas, VA, USA; A.T.T.C., code n: #CRL1446). Cells were grown in high-glucose Dulbecco’s modified Eagle’s medium (DMEM) (Euroclone, Pero, Italy) supplemented with 10% fetal bovine serum (FBS) (Euroclone), plus 2 mM glutamine(Euroclone), 100 IU/mL penicillin (Euroclone), and 100 IU/mL streptomycin (Euroclone) at 37 °C, 10% CO_2_. 

### 4.2. Measurement of Mitochondrial Respiration Rates

The respiratory rates were evaluated polarographically with a Clark-type oxygen electrode in a water-jacketed chamber (Hansatech Instruments, Norfolk, UK) at 37 °C, as described in [63]. Cells were collected via trypsinization, centrifuged and resuspended at 1–3 × 10^6^ cells/mL in 75 mM sucrose, 40 mM KCl, 5 mM KH_2_PO_4_, 3 mM MgCl_2_, 0.5 mM EDTA, 30 mM Tris–HCl, pH 7.4, plus 0.3 mM P1, P5-di(adenosine-5′) pentaphosphate (Ap5A) to avoid ADP dissipation by adenylate kinase. The cell suspension was transferred to the polarographic chamber, and an aliquot was utilized for cell counting and protein determination. After permeabilization by digitonin, substrates and inhibitors of mitochondrial respiratory chain complexes were added at the following concentrations: pyruvate (5 mM)/malate (2.5 mM), rotenone (200 nM), and succinate (5 mM). For OXPHOS efficiency measurement (P/O ratios), to induce transient stimulation of oxygen consumption, the substrates pyruvate/malate and succinate were followed by addition of 0.17 mM ADP and 0.08 mM ADP, respectively. The P/O was determined as the ratio between the nanomoles of added ADP and the atoms of oxygen consumed during the ADP-induced state 3 respiration [64]. The ATP production rates were determined by multiplying the values of the P/O ratio by double the corresponding ADP-stimulated (state 3) respiration rates [64].

### 4.3. Measurement of Mitochondrial Cellular ATP

The H9c2 cells were grown as described above. Once the cells were at 75% confluence, they were incubated for 30 min with 1 μM isoproterenol in the absence or presence of oligomycin (5 μM). After incubation, the cells were harvested and resuspended in phosphate-buffered saline, pH 7.4 (PBS). Next, 10^3^ cells were used to measure ATP level using a luciferin–luciferase reaction system (PROMEGA, Madison, WI, USA). Mitochondrial ATP level was determined as result of total ATP level minus ATP level in the presence of oligomycin.

### 4.4. Whole-Cell Lysate Preparation

The whole-cell lysate was prepared as described in [35]. Briefly, the cells were harvested from Petri dishes with 0.05% trypsin, 0.02% EDTA, pelleted by centrifugation at 500× *g* and then resuspended in PBS. The cell suspension was exposed to 1.7 mg of digitonin/mg cellular protein for 10 min on ice. The samples were then pelleted at 20,000× *g* and resuspended in PBS.

### 4.5. Measurements of Enzymatic Activities

The whole-cell lysates were exposed to ultrasound energy for 15 s at 0 °C.

The NADH-UQ oxidoreductase activity (complex I) was performed in 40 mM potassium phosphate buffer, pH 7.4, 5 Mm MgCl_2_, in the presence of 3 mM KCN, 1 μg/mL antimycin, 200 μM decylubiquinone, using 50 μg of mitoplast proteins, by following the oxidation of 100 μM NADH at 340–425 nm (Δε = 6.81 mM^−1^ cm^−1^). The activity was corrected for the residual activity measured in the presence of 1 μg/mL rotenone.

Cytochrome c oxidase (complex IV) activity was measured by following the oxidation of 10 μM ferrocytochrome c at 550–540 nm (Δε= 19.1 mM^−1^cm^−1^). Enzymatic activity was measured in 10 mM phosphate buffer, pH 7.4, using 20 μg of mitoplast proteins. This rate was inhibited over 95% by KCN (2 mM).

Ubiquinol–cytochrome *c* oxidoreductase (complex III) activity was measured at 550–540 nm (Δε = 19.1 mM^−1^ cm^−1^) as the initial rate of antimycin-sensitive cytochrome c reduction.

NADH-cytochrome *c* oxidoreductase (complex I + III) activity was measured at 550–540 nm (Δε = 19.1 mM^−1^ cm^−1^) as the initial rate of antimycin-sensitive cytochrome c reduction.

### 4.6. Electrophoretic Procedures and Western Blotting

Separation of respiratory complexes and their supercomplexes was performed by first dimension (1D) blue-native electrophoresis (BNE). Whole-cell lysates (150 µg) were suspended in 750mM aminocaproic acid, 50 mM Bis-Tris, 0.5 mM EDTA (mitochondrial buffer), plus 5:1 (mg/mg) digitonin. After 30 min on ice, the samples were centrifuged at 20,000× *g* for 30 min and the supernatant was recovered and kept on ice. The supernatants, representing the mitochondrial solubilized proteins, were loaded on 3–13% gradient native gel followed by the in-gel activity assays, performed as described in [65]. The 1D loading control was carried out by resuspending the mitochondrial solubilized proteins in laemmli lysis buffer and running the samples in SDS-PAGE, followed by immunoblotting with antibodies against TOM 20 (1:200 dilution, Santa Cruz Biotechnology, Dallas, TX, USA, code n. sc-11021) or TOM 70 (1:200 dilution, Santa Cruz, code n. sc.26495).

In order to dissociate the respiratory complexes and supercomplexes in their single subunits, each lane of 1D-BNE was subjected to second dimension (2D)-SDS PAGE. After the electrophoretic separation, the proteins were transferred to nitrocellulose and immunoblotted with antibodies against NDUFB6 (1:1000 dilution, Thermo Fisher–Invitrogen, code n. A21359, Waltham, MA, USA), NDUFA9 (1:500 dilution, Thermo Fisher–Invitrogen, code n. PA5-36993) and NDUFS4 (1:200 dilution, Thermo Fisher, code n. PA5-21677) subunits of complex I, Core II (1:1000 dilution, Thermo Fisher–Invitrogen, code n. 459220) subunit of complex III, Cox IV (1:1000 dilution, Thermo Fisher–Invitrogen, code n. A21348) subunit of complex IV and against β-ATPase (1:1000 dilution, Thermo Fisher–Invitrogen, code n. A21351) subunit of ATP synthase. The blots were incubated with HRP-conjugated secondary antibody generated in rabbit (1:5000) or mouse (1:5000) for 1 h at 4 °C. Densitometric analysis of discrete bands and spots was performed by Image Lab software (BioRad, Milan, Italy).

### 4.7. cDNA Construct and In Vitro Translation

Full-length human NDUFS4 and NDUFB11 cDNAs were generated by reverse transcriptase-PCR using RNA extracted from primary fibroblasts [30]. In vitro transcription/translation of cDNAs was performed in rabbit reticulocyte lysate (RRL) system (Promega Biotech, Madison, WI, USA). Essentially, 1 μg of construct was added to 50 μL Promega standard mixture containing T7 RNA polymerase and a standard amino acid mixture with [^35^S] Methionine/Cysteine (20 μCi). Incubation was performed at 30 °C for 90 min.

### 4.8. Rat Liver Mitochondria Isolation

RLM were isolated as described in [66]. Briefly, minced rat liver was homogenized in 0.22 M mannitol, 0.075 M sucrose, 1 mM EDTA, and 10 mM HEPES-KOH, pH 7.4 (HB) plus 0.25 mM phenylmethylsulfonyl fluoride as a protease inhibitor. The homogenate was centrifuged at 600× *g* for 10 min and the supernatant was further centrifuged at 4000× *g* for 10 min. The precipitate was resuspended in HB and centrifuged at 2000× *g* for 2 min plus 4000× *g* for 8 min. The mitochondrial pellet was resuspended in HB.

### 4.9. Import Assay

First, 5 µL of radioactive RRL translation mixture was added to 500 µg proteins of rat liver mitochondria in the import mixture containing: 210 mM mannitol, 0.35 mM MgCl2, 2.5 mg/mL BSA, 7 mM Hepes, pH 7.4, 3 mM ATP, 3 mM GTP, 15 mM malate, 30 mM pyruvate, and 1 mg/mL chloramphenicol; final volume 200 µL. The incubation was performed in the absence or in the presence of catalytic subunit of PKA (1 unit per 10 μg mitochondrial proteins) plus 7.5 mM sodium fluoride. After 1 h incubation at 30 °C, aliquots of the mixture were transferred to ice-cooled tubes and centrifuged at 4000× *g,* and mitochondrial proteins used for enzymatic activities, oxygen consumption analysis and BNE. For BNE, mitochondrial proteins were treated with 3.5 mg digitonin/mg protein for 20 min on ice. After that, solubilization proteins were pelleted at 20,000× *g* for 30 min at 4 °C. The resulting supernatants were resuspended in mitochondrial buffer and loaded on 3–13% gradient native gel, followed by autoradiography or immunoblotting or a second dimension in 12% SDS-PAGE. Radioactive protein bands were detected by Personal FX at “phosphorus imager” (Bio-Rad, Milan, Italy) and quantified by VERSADOC (Bio-Rad). Immuno-revealed bands were quantified by densitometric analysis using Image Lab system (Bio-Rad).

## Supplementary Materials

The following supporting information can be downloaded at: https://www.mdpi.com/article/10.3390/ijms23179655/s1.

## References

[1] Efremov R.G., Baradaran R., Sazanov L.A. (2010). The Architecture of Respiratory Complex I. Nature.

[2] Papa S., Martino P.L., Capitanio G., Gaballo A., De Rasmo D., Signorile A., Petruzzella V. (2012). The Oxidative Phosphorylation System in Mammalian Mitochondria. Adv. Exp. Med. Biol..

[3] Schägger H., Pfeiffer K. (2000). Supercomplexes in the Respiratory Chains of Yeast and Mammalian Mitochondria. EMBO J..

[4] Rugolo M., Zanna C., Ghelli A.M. (2021). Organization of the Respiratory Supercomplexes in Cells with Defective Complex III: Structural Features and Metabolic Consequences. Life.

[5] Cogliati S., Cabrera-Alarcón J.L., Enriquez J.A. (2021). Regulation and Functional Role of the Electron Transport Chain Supercomplexes. Biochem. Soc. Trans..

[6] Guarás A., Perales-Clemente E., Calvo E., Acín-Pérez R., Loureiro-Lopez M., Pujol C., Martínez-Carrascoso I., Nuñez E., García-Marqués F., Rodríguez-Hernández M.A. (2016). The CoQH2/CoQ Ratio Serves as a Sensor of Respiratory Chain Efficiency. Cell Rep..

[7] Speijer D. (2019). Can All Major ROS Forming Sites of the Respiratory Chain Be Activated By High FADH2/NADH Ratios?. BioEssays.

[8] Maranzana E., Barbero G., Falasca A.I., Lenaz G., Genova M.L. (2013). Mitochondrial Respiratory Supercomplex Association Limits Production of Reactive Oxygen Species from Complex I. Antioxid. Redox Signal..

[9] Lopez-Fabuel I., Le Douce J., Logan A., James A.M., Bonvento G., Murphy M.P., Almeida A., Bolaños J.P. (2016). Complex I Assembly into Supercomplexes Determines Differential Mitochondrial ROS Production in Neurons and Astrocytes. Proc. Natl. Acad. Sci. USA.

[10] Calvo E., Cogliati S., Hernansanz-Agustín P., Loureiro-López M., Guarás A., Casuso R.A., García-Marqués F., Acín-Pérez R., Martí-Mateos Y., Silla-Castro J.C. (2020). Functional Role of Respiratory Supercomplexes in Mice: SCAF1 Relevance and Segmentation of the Qpool. Sci. Adv..

[11] Acín-Pérez R., Bayona-Bafaluy M.P., Fernández-Silva P., Moreno-Loshuertos R., Pérez-Martos A., Bruno C., Moraes C.T., Enríquez J.A. (2004). Respiratory Complex III Is Required to Maintain Complex I in Mammalian Mitochondria. Mol. Cell.

[12] Schägger H., de Coo R., Bauer M.F., Hofmann S., Godinot C., Brandt U. (2004). Significance of Respirasomes for the Assembly/Stability of Human Respiratory Chain Complex I*. J. Biol. Chem..

[13] Moreno-Lastres D., Fontanesi F., García-Consuegra I., Martín M.A., Arenas J., Barrientos A., Ugalde C. (2012). Mitochondrial Complex I Plays an Essential Role in Human Respirasome Assembly. Cell Metab..

[14] Lobo-Jarne T., Pérez-Pérez R., Fontanesi F., Timón-Gómez A., Wittig I., Peñas A., Serrano-Lorenzo P., García-Consuegra I., Arenas J., Martín M.A. (2020). Multiple Pathways Coordinate Assembly of Human Mitochondrial Complex IV and Stabilization of Respiratory Supercomplexes. EMBO J..

[15] Protasoni M., Pérez-Pérez R., Lobo-Jarne T., Harbour M.E., Ding S., Peñas A., Diaz F., Moraes C.T., Fearnley I.M., Zeviani M. (2020). Respiratory Supercomplexes Act as a Platform for Complex III-Mediated Maturation of Human Mitochondrial Complexes I and IV. EMBO J..

[16] Cocco T., Pacelli C., Sgobbo P., Villani G. (2009). Control of OXPHOS Efficiency by Complex I in Brain Mitochondria. Neurobiol. Aging.

[17] Carroll J., Fearnley I.M., Skehel J.M., Shannon R.J., Hirst J., Walker J.E. (2006). Bovine Complex I Is a Complex of 45 Different Subunits. J. Biol. Chem..

[18] Kampjut D., Sazanov L.A. (2022). Structure of Respiratory Complex I—An Emerging Blueprint for the Mechanism. Curr. Opin. Struct. Biol..

[19] Guerrero-Castillo S., Baertling F., Kownatzki D., Wessels H.J., Arnold S., Brandt U., Nijtmans L. (2017). The Assembly Pathway of Mitochondrial Respiratory Chain Complex I. Cell Metab..

[20] Wong H.-S., Dighe P.A., Mezera V., Monternier P.-A., Brand M.D. (2017). Production of Superoxide and Hydrogen Peroxide from Specific Mitochondrial Sites under Different Bioenergetic Conditions. J. Biol. Chem..

[21] Papa S., De Rasmo D., Scacco S., Signorile A., Technikova-Dobrova Z., Palmisano G., Sardanelli A.M., Papa F., Panelli D., Scaringi R. (2008). Mammalian Complex I: A Regulable and Vulnerable Pacemaker in Mitochondrial Respiratory Function. Biochim. Biophys. Acta.

[22] Padavannil A., Ayala-Hernandez M.G., Castellanos-Silva E.A., Letts J.A. (2021). The Mysterious Multitude: Structural Perspective on the Accessory Subunits of Respiratory Complex I. Front. Mol. Biosci..

[23] Cortés-Rojo C., Vargas-Vargas M.A., Olmos-Orizaba B.E., Rodríguez-Orozco A.R., Calderón-Cortés E. (2020). Interplay between NADH Oxidation by Complex I, Glutathione Redox State and Sirtuin-3, and Its Role in the Development of Insulin Resistance. Biochim. Biophys. Acta Mol. Basis Dis..

[24] Papa S., De Rasmo D. (2013). Complex I Deficiencies in Neurological Disorders. Trends Mol. Med..

[25] Papa S., Scacco S., De Rasmo D., Signorile A., Papa F., Panelli D., Nicastro A., Scaringi R., Santeramo A., Roca E. (2010). CAMP-Dependent Protein Kinase Regulates Post-Translational Processing and Expression of Complex I Subunits in Mammalian Cells. Biochim. Biophys. Acta.

[26] Signorile A., Micelli L., De Rasmo D., Santeramo A., Papa F., Ficarella R., Gattoni G., Scacco S., Papa S. (2014). Regulation of the Biogenesis of OXPHOS Complexes in Cell Transition from Replicating to Quiescent State: Involvement of PKA and Effect of Hydroxytyrosol. Biochim. Biophys. Acta.

[27] Bellomo F., Piccoli C., Cocco T., Scacco S., Papa F., Gaballo A., Boffoli D., Signorile A., D’Aprile A., Scrima R. (2006). Regulation by the CAMP Cascade of Oxygen Free Radical Balance in Mammalian Cells. Antioxid. Redox Signal..

[28] De Rasmo D., Gattoni G., Papa F., Santeramo A., Pacelli C., Cocco T., Micelli L., Sardaro N., Larizza M., Scivetti M. (2011). The β-Adrenoceptor Agonist Isoproterenol Promotes the Activity of Respiratory Chain Complex I and Lowers Cellular Reactive Oxygen Species in Fibroblasts and Heart Myoblasts. Eur. J. Pharmacol..

[29] De Rasmo D., Signorile A., Larizza M., Pacelli C., Cocco T., Papa S. (2012). Activation of the CAMP Cascade in Human Fibroblast Cultures Rescues the Activity of Oxidatively Damaged Complex I. Free Radic. Biol. Med..

[30] De Rasmo D., Panelli D., Sardanelli A.M., Papa S. (2008). CAMP-Dependent Protein Kinase Regulates the Mitochondrial Import of the Nuclear Encoded NDUFS4 Subunit of Complex I. Cell. Signal..

[31] Lazarou M., McKenzie M., Ohtake A., Thorburn D.R., Ryan M.T. (2007). Analysis of the Assembly Profiles for Mitochondrial- and Nuclear-DNA-Encoded Subunits into Complex I. Mol. Cell. Biol..

[32] Calvaruso M.A., Willems P., van den Brand M., Valsecchi F., Kruse S., Palmiter R., Smeitink J., Nijtmans L. (2012). Mitochondrial Complex III Stabilizes Complex I in the Absence of NDUFS4 to Provide Partial Activity. Hum. Mol. Genet..

[33] Budde S.M., van den Heuvel L.P., Janssen A.J., Smeets R.J., Buskens C.A., DeMeirleir L., Van Coster R., Baethmann M., Voit T., Trijbels J.M. (2000). Combined Enzymatic Complex I and III Deficiency Associated with Mutations in the Nuclear Encoded NDUFS4 Gene. Biochem. Biophys. Res. Commun..

[34] Suthammarak W., Somerlot B.H., Opheim E., Sedensky M., Morgan P.G. (2013). Novel Interactions between Mitochondrial Superoxide Dismutases and the Electron Transport Chain. Aging Cell.

[35] Timón-Gómez A., Pérez-Pérez R., Nyvltova E., Ugalde C., Fontanesi F., Barrientos A. (2020). Protocol for the Analysis of Yeast and Human Mitochondrial Respiratory Chain Complexes and Supercomplexes by Blue Native Electrophoresis. STAR Protoc..

[36] De Rasmo D., Palmisano G., Scacco S., Technikova-Dobrova Z., Panelli D., Cocco T., Sardanelli A.M., Gnoni A., Micelli L., Trani A. (2010). Phosphorylation Pattern of the NDUFS4 Subunit of Complex I of the Mammalian Respiratory Chain. Mitochondrion.

[37] Budde S.M.S., van den Heuvel L.P.W.J., Smeitink J.a.M. (2002). The Human Complex I NDUFS4 Subunit: From Gene Structure to Function and Pathology. Mitochondrion.

[38] Raha S., Myint A.T., Johnstone L., Robinson B.H. (2002). Control of Oxygen Free Radical Formation from Mitochondrial Complex I: Roles for Protein Kinase A and Pyruvate Dehydrogenase Kinase. Free Radic. Biol. Med..

[39] Jimenez-Blasco D., Busquets-Garcia A., Hebert-Chatelain E., Serrat R., Vicente-Gutierrez C., Ioannidou C., Gómez-Sotres P., Lopez-Fabuel I., Resch-Beusher M., Resel E. (2020). Glucose Metabolism Links Astroglial Mitochondria to Cannabinoid Effects. Nature.

[40] Chen R., Fearnley I.M., Peak-Chew S.Y., Walker J.E. (2004). The Phosphorylation of Subunits of Complex I from Bovine Heart Mitochondria. J. Biol. Chem..

[41] Papa S., Scacco S., Sardanelli A.M., Vergari R., Papa F., Budde S., van den Heuvel L., Smeitink J. (2001). Mutation in the NDUFS4 Gene of Complex I Abolishes CAMP-Dependent Activation of the Complex in a Child with Fatal Neurological Syndrome. FEBS Lett..

[42] Lapuente-Brun E., Moreno-Loshuertos R., Acín-Pérez R., Latorre-Pellicer A., Colás C., Balsa E., Perales-Clemente E., Quirós P.M., Calvo E., Rodríguez-Hernández M.A. (2013). Supercomplex Assembly Determines Electron Flux in the Mitochondrial Electron Transport Chain. Science.

[43] Genova M.L., Lenaz G. (2014). Functional Role of Mitochondrial Respiratory Supercomplexes. Biochim. Biophys. Acta.

[44] Scacco S., Petruzzella V., Budde S., Vergari R., Tamborra R., Panelli D., van den Heuvel L.P., Smeitink J.A., Papa S. (2003). Pathological Mutations of the Human NDUFS4 Gene of the 18-KDa (AQDQ) Subunit of Complex I Affect the Expression of the Protein and the Assembly and Function of the Complex. J. Biol. Chem..

[45] Ugalde C., Janssen R.J.R.J., van den Heuvel L.P., Smeitink J.A.M., Nijtmans L.G.J. (2004). Differences in Assembly or Stability of Complex I and Other Mitochondrial OXPHOS Complexes in Inherited Complex I Deficiency. Hum. Mol. Genet..

[46] Kruse S.E., Watt W.C., Marcinek D.J., Kapur R.P., Schenkman K.A., Palmiter R.D. (2008). Mice with Mitochondrial Complex I Deficiency Develop a Fatal Encephalomyopathy. Cell Metab..

[47] Acín-Pérez R., Fernández-Silva P., Peleato M.L., Pérez-Martos A., Enriquez J.A. (2008). Respiratory Active Mitochondrial Supercomplexes. Mol. Cell.

[48] Kolata P., Efremov R.G. (2021). Structure of Escherichia Coli Respiratory Complex I Reconstituted into Lipid Nanodiscs Reveals an Uncoupled Conformation. eLife.

[49] Prlic A., Bliven S., Rose P.W., Bluhm W.F., Bizon C., Godzik A., Bourne P.E. (2010). Pre-Calculated Protein Structure Alignments at the RCSB PDB Website. Bioinformatics.

[50] Li Z., Jaroszewski L., Iyer M., Sedova M., Godzik A. (2020). FATCAT 2.0: Towards a Better Understanding of the Structural Diversity of Proteins. Nucleic Acids Res..

[51] Humphrey W., Dalke A., Schulten K. (1996). VMD: Visual Molecular Dynamics. J. Mol. Graph..

[52] Frishman D., Argos P. (1995). Knowledge-Based Protein Secondary Structure Assignment. Proteins.

[53] Burley S.K., Berman H.M., Bhikadiya C., Bi C., Chen L., Di Costanzo L., Christie C., Dalenberg K., Duarte J.M., Dutta S. (2019). RCSB Protein Data Bank: Biological Macromolecular Structures Enabling Research and Education in Fundamental Biology, Biomedicine, Biotechnology and Energy. Nucleic Acids Res..

[54] Agip A.-N.A., Blaza J.N., Bridges H.R., Viscomi C., Rawson S., Muench S.P., Hirst J. (2018). Cryo-EM Structures of Complex I from Mouse Heart Mitochondria in Two Biochemically Defined States. Nat. Struct. Mol. Biol..

[55] Bridges H.R., Fedor J.G., Blaza J.N., Di Luca A., Jussupow A., Jarman O.D., Wright J.J., Agip A.-N.A., Gamiz-Hernandez A.P., Roessler M.M. (2020). Structure of Inhibitor-Bound Mammalian Complex I. Nat. Commun..

[56] Chung I., Serreli R., Cross J.B., Di Francesco M.E., Marszalek J.R., Hirst J. (2021). Cork-in-Bottle Mechanism of Inhibitor Binding to Mammalian Complex I. Sci. Adv..

[57] Chung I., Wright J.J., Bridges H.R., Ivanov B.S., Biner O., Pereira C.S., Arantes G.M., Hirst J. (2022). Cryo-EM Structures Define Ubiquinone-10 Binding to Mitochondrial Complex I and Conformational Transitions Accompanying Q-Site Occupancy. Nat. Commun..

[58] Yin Z., Burger N., Kula-Alwar D., Aksentijević D., Bridges H.R., Prag H.A., Grba D.N., Viscomi C., James A.M., Mottahedin A. (2021). Structural Basis for a Complex I Mutation That Blocks Pathological ROS Production. Nat. Commun..

[59] Grba D.N., Blaza J.N., Bridges H.R., Agip A.-N.A., Yin Z., Murai M., Miyoshi H., Hirst J. (2022). Cryo-Electron Microscopy Reveals How Acetogenins Inhibit Mitochondrial Respiratory Complex I. J. Biol. Chem..

[60] Gu J., Liu T., Guo R., Zhang L., Yang M. (2022). The Coupling Mechanism of Mammalian Mitochondrial Complex I. Nat. Struct. Mol. Biol..

[61] Guo R., Zong S., Wu M., Gu J., Yang M. (2017). Architecture of Human Mitochondrial Respiratory Megacomplex I2III2IV2. Cell.

[62] Piccoli C., Scacco S., Bellomo F., Signorile A., Iuso A., Boffoli D., Scrima R., Capitanio N., Papa S. (2006). CAMP Controls Oxygen Metabolism in Mammalian Cells. FEBS Lett..

[63] Sgobbo P., Pacelli C., Grattagliano I., Villani G., Cocco T. (2007). Carvedilol Inhibits Mitochondrial Complex I and Induces Resistance to H2O2 -Mediated Oxidative Insult in H9C2 Myocardial Cells. Biochim. Biophys. Acta.

[64] Chance B., Williams G.R. (1955). Respiratory Enzymes in Oxidative Phosphorylation. I. Kinetics of Oxygen Utilization. J. Biol. Chem..

[65] Nijtmans L.G.J., Henderson N.S., Holt I.J. (2002). Blue Native Electrophoresis to Study Mitochondrial and Other Protein Complexes. Methods.

[66] Ito A., Ogishima T., Ou W., Omura T., Aoyagi H., Lee S., Mihara H., Izumiya N. (1985). Effects of Synthetic Model Peptides Resembling the Extension Peptides of Mitochondrial Enzyme Precursors on Import of the Precursors into Mitochondria. J. Biochem..

