# Supplementary material for: cAMP/PKA Signaling Modulates Mitochondrial Supercomplex Organization
Source: Int J Mol Sci. 2022 Aug 25;23(17):9655. doi: 10.3390/ijms23179655 (PMC9455794; doi:10.3390/ijms23179655)
Supplement: Supplementary file 1 [file ijms-23-09655-s001.zip › ijms-1874424-supplementary.pdf]

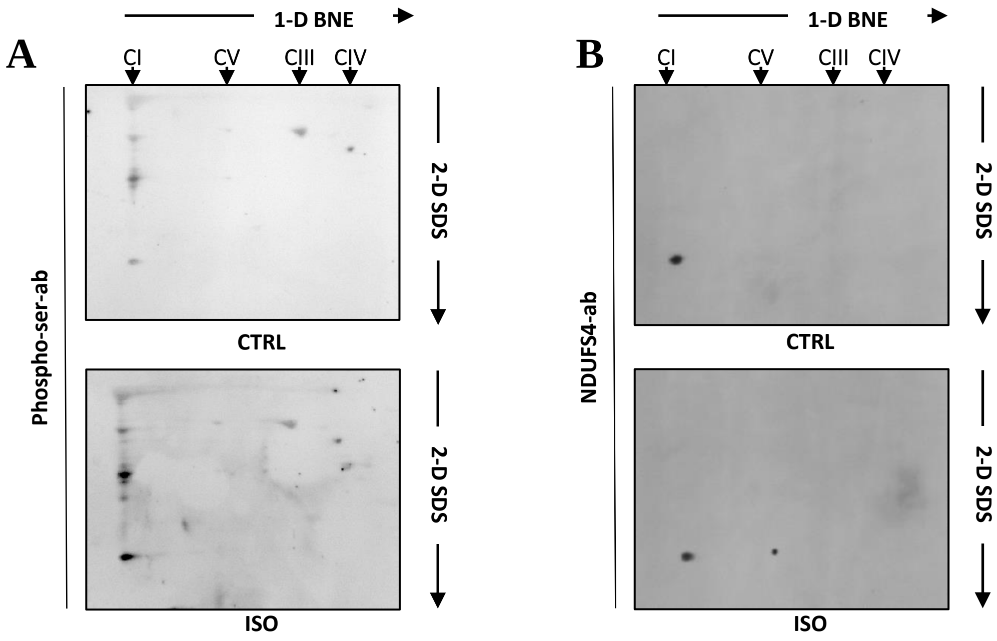

Supplemental Figure S1. Rat liver was shredded and incubated in an isotonic buffer in the absence (CTRL) or in the presence of 100 µM isoproterenol (ISO). After 30 minutes of incubation, mitochondria were isolated as described in material and methods section and subjected to BNE/SDS. For BNE, in order to completely separate the individual free complexes of OXPHOS, the mitochondria were treated with 0.4% n-dodecyl-beta-maltoside. After the electrophoretic separation the proteins were transferred to nitrocellulose and immunoblotted with antibodies against phospho-serine (A) and NDUFS4 subunits of complex I (B).

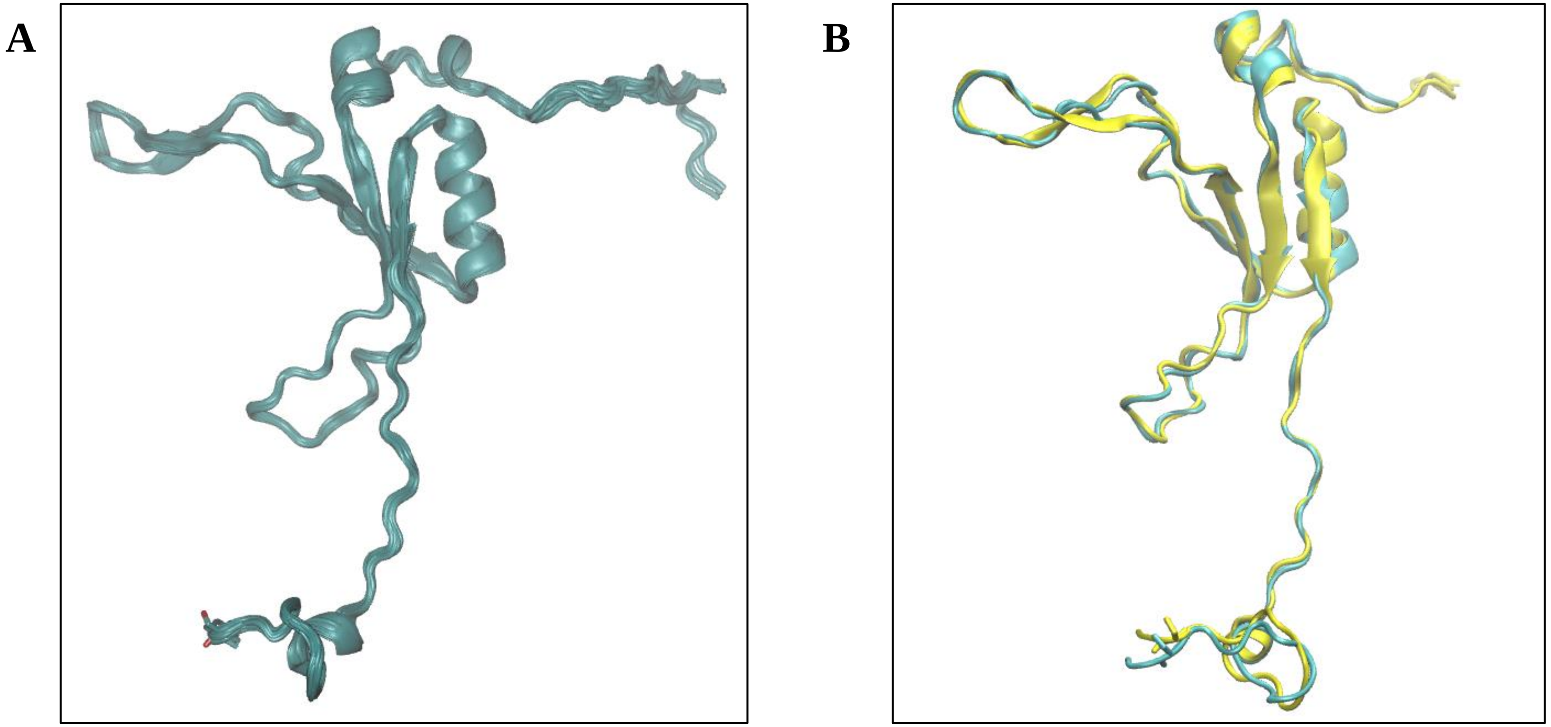

Supplemental Figure S2. Protein structure overlap. The atomic coordinates of the NDUFS4 subunits were obtained from the PDB [52]. The codes of the structures used were: 6G2J, 6ZK9, 6ZKC, 6ZKD, 6ZKE, 6ZKF, 6ZKG, 6ZKH, 6ZKI, 6ZKJ, 6ZKL, 6ZKM, 6ZKN, 6ZKO, 6ZKP, 6ZKQ, 6ZKS, 6ZKU, 6ZKV, 6ZR2, 6ZTQ, 7AK5, 7B93, 7PSA, 7QSD, 7QSK, 7QSL, 7QSM, 7QSN, 7QSO, 7V2C, 7V2D, 7V2E, 7V2F, 7V2H, 7V2K, 7V2R, 7V30, 7V31, 7V32, 7V33, 7V3M, 7VB7, 7VBN, 7VBZ, 7VWJ [18,53–59]. The NDUFS4 coordinates in the human supercomplex (PDB entry 5XTH) are reported in [60]. Rigid structural superposition has been performed as described [48,49] using the NDUFS4 in the PDB entry 6ZKO as reference and visualized in VMD [50]. Secondary structure was determined as described in [51]. A, Overlap of the mammalian NDUFS4 structures at resolution of 3.40 Å or better deposited in the PDB (see above for the PDB codes). For a single structure, the serine in the amino terminal sequence RVSTK is reported in licorice. B, Overlap of NDUFS4 structures in a super complex (5XTH in cyan) and in an isolated complex (6ZK9 in yellow). Serine in the amino terminal sequence RVSTK is reported in licorice in both structures. The distance between the oxygen atoms of the two serines in the reported alignment is about 2.4 Å, but the resolution of the structures is 3.90 Å for 5XTH and 2.30 Å for 6ZK9 (chosen as the reference high resolution structure).

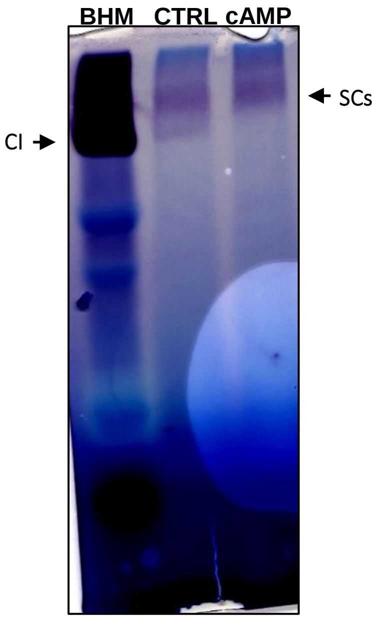

Supplemental Figure S3. Neonatal normal human dermal fibroblasts (NHDF-neo, Cambrex #CC-2509, East Rutherford, NJ, USA) were grown in the exponential phase in high glucose Dulbecco's modified Eagle's medium (DMEM) (EuroClone, Paignton, UK) supplemented with 10% fetal bovine serum (FBS), plus 2 mM glutamine, 100 IU/ml penicillin and 100 IU/ml streptomycin (Euroclone, Paignton, UK) at 37 °C, 5% CO2. Once to 90% confluence, cells were collected and permeabilized by digitonin (25 µg digitonine/10^6 cells). Then, RRL system, with newly synthesized [^{35}S]met-labelled NDUFS4 in the absence (CTRL) or in presence (cAMP) of 100 µM cAMP, was added to the mixture. After 30 minutes incubation at 37°C, cells were spun down at 10000 xg, resuspended in mitochondrial buffer and treated for 10 minutes in ice in the presence of a of n-dodecyl-beta-maltoside (1.5%). The samples were then centrifuged at 20000 xg and the supernantants subjected to BNE followed by complex I in gel activity assay. The first lane reppresents a control for molecular weight of free complex I in bovine heart mitochondria (BHM).
